# Bone Quality in Resorbed Posterior Maxilla Affects Osteogenesis After Sinus Floor Augmentation: A Retrospective Analysis

**DOI:** 10.1016/j.identj.2025.109397

**Published:** 2026-01-30

**Authors:** Wenjie Zhou, Shengchi Fan, Emilio A. Cafferata, Yihan Shen, Yiqun Wu

**Affiliations:** a Department of Second Dental Center, Shanghai Ninth People's Hospital, Shanghai Jiao Tong University School of Medicine; College of Stomatology, Shanghai Jiao Tong University; National Center for Stomatology; National Clinical Research Center for Oral Diseases; Shanghai Key Laboratory of Stomatology; Research Unit of Oral and Maxillofacial Regenerative Medicine, Chinese Academy of Medical Sciences, Shanghai, China; b Department of Oral Surgery and Implantology, Goethe University, Carolinum, Frankfurt am Main, Germany; c Oral Surgery and Implantology, Faculty of Medicine and Health Sciences, University of Barcelona, Barcelona, Spain; d Department of Oral and Maxillofacial Surgery, Plastic Operations, University Medical Center Mainz, Mainz, Germany; e Oral Peri-Implant Research Group, School of Dentistry, Universidad Científica del Sur, Lima, Perú

**Keywords:** Biopsy, Bone density, Cone-beam computer tomography, Histology, Posterior maxilla, Sinus floor augmentation

## Abstract

**Introduction and Aims:**

Compromised bone quality and quantity in the posterior maxilla are widely recognised as significant risk factors for implant failures. This study aimed to (1) assess the bone quality of the resorbed posterior maxilla both radiographically and histologically and (2) evaluate the impact of native alveolar bone quality on osteogenesis following maxillary sinus floor augmentation (MSFA).

**Methods:**

Patients presenting advanced posterior maxillary atrophy (residual bone height ≤ 4 mm) underwent MSFA via a lateral window approach using deproteinised bovine bone matrix (DBBM). Bone core biopsies were collected during second-stage implant placement for histological analyses. Preoperative cone-beam computer tomography (CBCT) images were used to classify bone quality based on cortical bone configuration. Multiple linear regression was employed to identify factors influencing osteogenesis.

**Results:**

A total of 190 sinuses from 176 patients (96 males/80 females, mean age: 45.77 ± 4.13 years) underwent MSFA and biopsy. Radiographic analysis revealed the presence of bicortical bone in 42.21% of the maxillary ridges, unicortical bone in 25.97%, no cortical bone in 19.48% and sinus floor–crestal bone fusion in 12.34%. Histologically, native bone comprised 40.01% ± 14.85% of mineralised trabeculae while newly formed bone accounted for 18.77% ± 5.69% in the DBBM grafted areas. A significant positive correlation was observed between native bone trabecula percentage and new bone formation (r = 0.23, *P* = .04). No significant associations were found with age, sex, healing time or radiographic alveolar type (*P* > .05).

**Conclusion:**

Greater density of native trabecular bone may contribute to enhanced osteogenesis following MSFA.

**Clinical relevance:**

This study enhances our understanding of the bone characteristics of the resorbed posterior maxilla. It emphasises the need to consider the bone quality of the recipient site before conducting a MSFA for determining the surgical procedure and predicting the prognosis.

## Introduction

The early loss of posterior maxillary teeth, resulting in progressive alveolar ridge resorption and sinus pneumatisation, often leads to severe maxillary atrophy.^1^ Indeed, this bidirectional reduction of bone vertical dimension, along with limited bone quality, makes optimal dental implant positioning and restoration challenging.^1^^,^^2^ Moreover, the limited bone quality and quantity have consistently been considered as major risk factors for implant failure in this region, highlighting the importance of a thorough implant site development assessment to secure favourable treatment prognosis.

Bone quality is a key factor affecting the bone-to-implant contact (BIC), a major determinant of successful implant osseointegration.^3^ From a surgical perspective, achieving implant primary stability depends on bone quality, which further influences prosthodontic decisions, such as the timing and approach for implant loading.^4^ In addition, recent studies have reported that bone quality influences the accuracy of computer-assisted guided implant surgery.^5^, ^6^, ^7^, ^8^ Therefore, these studies underscore the significance of evaluating alveolar bone quality for clinical decision-making, from implant placement and loading protocols to implant selection and guidance approaches for computer-guided surgery.

Traditionally, maxilla bone quality has been assessed by classifying them into 4 types, using either the Lekholm and Zarb’s^9^ or the Misch’s classifications.^10^ Accordingly, the posterior maxilla is mostly composed of type 3 or 4 (Lekholm and Zarb’s classification) or D3 or D4 bone (Misch’s classification) because of its thin cortical bone layer and the underlying highly porous trabecular bone. However, both classifications intended to determine the proportion of cortical and trabecular bone are based on 2-dimensional radiographic evaluation and tactile feedback during implant osteotomies, which can be inconsistent and lacking scientific validation. Specifically, (1) the broad definitions given by these classifications may lead to a lack of standardisation across studies, hindering their comparability and clinical application of results^11^; and (2) the outcomes of either the radiological or the tactile assessments are subject to the operator’s experience, introducing subjectivity and making reproducibility difficult.^12^ By contrast, histological examination of bone biopsy specimens remains the most diverse and reliable method for the assessment of bone structures.

Lindhe et al. reported that in fully healed edentulous maxillae, more than 50% of the bone is mineralised, with the remainder consisting of osteoid matrix, bone marrow, fibrous tissue and other components.^13^^,^^14^ The native bone from the residual alveolar ridge and the sinus floor represents a critical source of blood supply and mesenchymal stem cells needed for bone regeneration. Despite these insights, studies specifically assessing the bone quality in the resorbed posterior maxilla are scarce, and even fewer have explored whether this bone quality influences osteogenesis following maxillary sinus floor augmentation (MSFA). Therefore, this study aimed to evaluate the bone quality of the resorbed posterior maxilla both radiographically and histologically, and to investigate the effect of native bone quality on osteogenesis after MSFA.

## Material and methods

### Study design and population

This retrospective study protocol was approved by the Institutional Review Board of Shanghai Ninth People's Hospital, China (No: 2015[78] and SH9H-2021-T63-1) and was conducted in compliance with the Helsinki Declaration, as revised in 2013, and reported following the STROBE guidelines for observational studies.^15^

All patients referred to the Department of Oral Implantology or the Second Dental Center, Ninth People’s Hospital, Shanghai Jiao Tong University, School of Medicine, between June 2015 to September 2021, with an indication for implant rehabilitation of the atrophic posterior maxilla were consecutively screened for eligibility.

Eligible patients were adults (≥18 years) presenting maxillary molar(s) or premolar(s) loss ≥3 months, and residual bone height (RBH) ≤ 4 mm and adequate bone width at the target edentulous site, who provided signed informed consent for participation. Exclusion criteria included active sinus infection, uncontrolled inflammatory diseases—such as uncontrolled periodontal disease and uncontrolled diabetes—heavy smoking (>10 cigarettes/day), history of head/neck radiation therapy (>60 Gy doses), osteoporosis or bisphosphonates/steroids therapy, and incomplete records.

### Surgical procedures

#### MSFA via lateral window approach

All surgeries were performed by a single experienced surgeon (Y.W.). Briefly, a full-thickness mucoperiosteal flap exposed the maxillary lateral wall, and then an osteotomy window was prepared to access the sinus cavity using piezoelectric instruments. The Schneiderian membrane was gently lifted from the sinus floor and elevated, creating a space that was subsequently grafted with deproteinised bovine bone matrix (DBBM) granules (Bio-Oss). Afterwards, a native collagen membrane (Bio-Gide) was placed to cover the lateral window, followed by flap repositioning and suture, achieving primary closure.

#### Staged implant placement and biopsy

After a healing period of 4-12 months, bone core specimens were harvested at grafted sites with a trephine drill (outer diameter 4.0 mm, inner diameter 3.0 mm, length 10 mm), followed by twist drills as recommended by the manufacturer. Then, 10-12 mm tissue level implants (Straumann) were inserted. Biopsied tissues were immediately fixed in 10% neutral buffered formalin. When multiple implants were indicated, a single bone specimen was taken from the implant site with the lowest RBH.

### Radiographic analysis

Cone-beam computed tomography (CBCT) scans were acquired within 1 month prior to the MSFA procedures. CBCT images were obtained using the Planmeca Promax Tomography System with the following operating parameters: 5 mA, 96 kV; voxel size: 0.2 mm; field of view: 13 cm × 9 cm. The primary data were exported as DICOM files and reconstructed using implant planning software (coDiagnostiX).

**Fig. 1:**
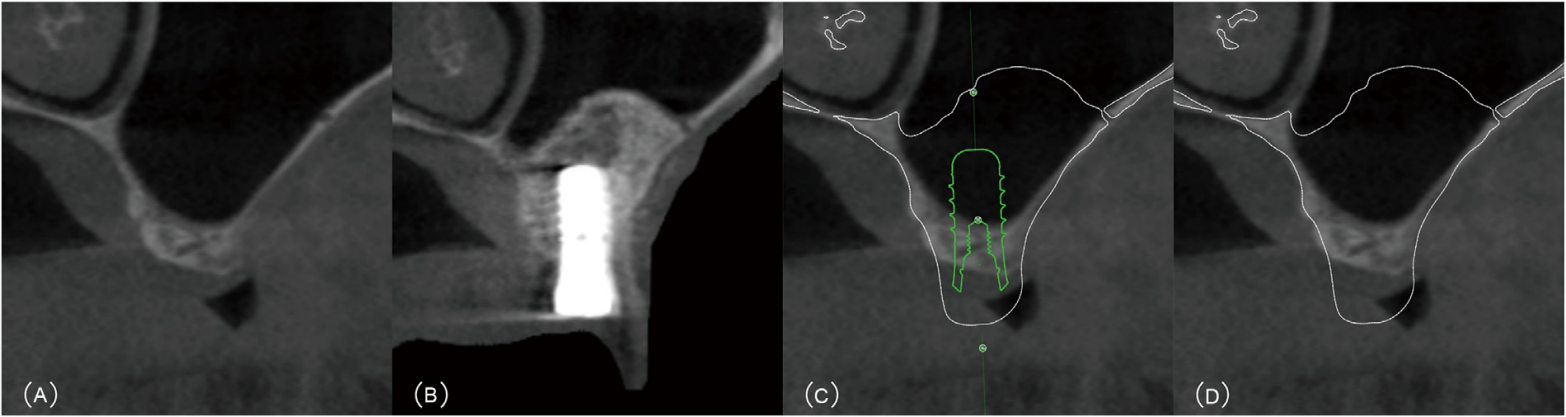
Determination of the assessment region on the CBCT image. A, Sagittal section of the preoperative CBCT showing the posterior maxillary ridge. B, Sagittal section of the postoperative CBCT showing the posterior maxillary ridge after implant placement and MSFA. C, Information on the implant, grafting bone and maxillary jaw was extracted from the postoperative CBCT data and then superimposed with the preoperative CBCT. D, The virtual implant was hidden to clearly expose the region for assessment.

**Fig. 2:**
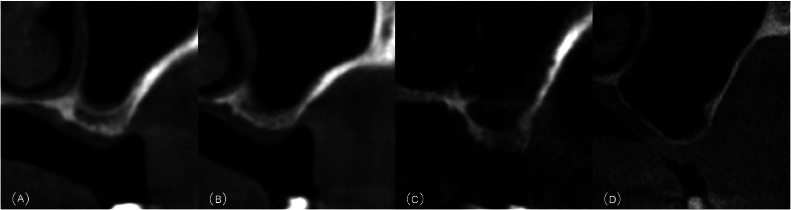
Classification of bone quality in the posterior maxilla according to the presence of cortication of the sinus floor and crestal bone. A, Type a: bicortical bone. B, Type b: unicortial bone. C, Type c: no cortical bone. D, Type d: Fusion of the sinus floor bone and native crestal bone.

For each patient, a digital wax-up and surgical planning were performed prior to the surgery, following the protocol described by Zhou et al.^16^ Radiographic measurements were taken from sagittal sections at the planned implant sites, according to pre-prosthetic planning (Figure 1). RBH was defined as the vertical distance from the alveolar crest to the sinus floor, measured along the long axis of the virtual implant. The bone quality of the maxillary ridge was assessed and classified using a modified version of the classification proposed by Choucroun et al.^17^ (Figure 2).

### Histological and histomorphometric analysis

Specimens were decalcified, paraffin embedded, and sectioned 6 μm thick along the longitudinal axis for haematoxylin and eosin (H&E) staining. For each specimen, 3 sections (#10, #20, #30) were selected and examined under a light microscope. The native bone and grafted bone areas were identified based on their morphological characteristics, by an experienced examiner. For each section, an overview image was captured at ×4 magnification, and the native bone area was then cropped from the image. Afterwards, an additional image was acquired at ×10 magnification from a randomly selected region within the grafted bone area.

All images were imported into the Image Pro-Plus software (version 6.0) for quantitative histomorphometry. Within each image, the regions of interest—native bone trabeculae in the native bone area and the newly formed bone in the grafted bone area—were automatically delineated by a colour-contrast algorithm and subsequently tuned manually. The native bone trabeculae and newly formed bone area percentages were calculated as the number of selected pixels divided by the total pixel count. The reported results represent the mean independent measurements obtained from 3 sections of each specimen.

### Data analysis

Two examiners (W.Z. and Y.S.), not involved in patient treatment and each with more than 5 years of experience, independently evaluated the radiological and histomorphometric images/data. The inter-examiner agreement was assessed using the Kappa test for categorical variables (radiographical alveolar type). The inter-rater reliability was determined with the interclass correlation coefficient (ICC) for continuous variables (% of native bone trabeculae and % of newly formed bone). All measurements were analysed at the sinus and biopsy levels.

To investigate the association between radiographic and histomorphometric outcomes of bone quality, one-way ANOVA was used to analyse the mean percentage of native bone trabecula from the specimens with the radiographic classification of the resorbed posterior maxilla. A multiple linear regression model, with r values as correlation coefficients, assessed the associations between histomorphometric outcomes (mean % of native bone trabecula and newly formed bone), healing time and radiographic alveolar type. Generalised estimating equations (GEE) analysis was performed using baseline variables (age and gender) as predictors for the percentage of newly formed bone while adjusting for patients who had bilateral MSFA. Age (continuous) and gender (binary) were included as fixed effects, and an exchangeable correlation structure was used to model the relationship between surgeries on the same patient. Statistical significance was set at *P* < .05. All analyses were performed using the PASW Statistics 18.0 software program.

## Results

### Patient’s characteristics

A total of 176 patients, corresponding to 190 maxillary sinuses, underwent MSFA via a lateral approach, with bone biopsies obtained at the implant sites. The study sample comprised 96 males and 80 females (mean age: 45.77 ± 4.13 years; range: 20-72 years). The mean healing time after MSFA was 8.14 ± 4.77 months (range: 4.0-12.4 months).

**Fig. 3:**
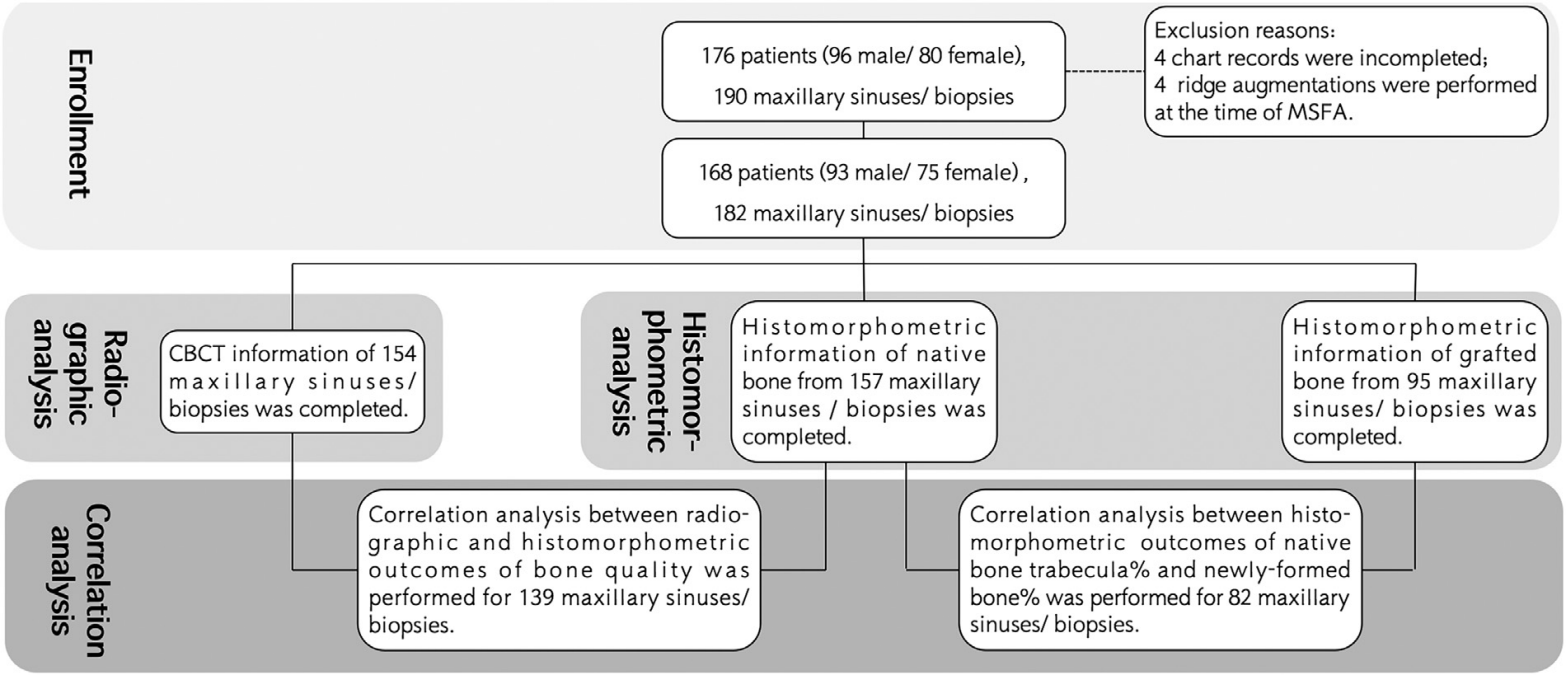
A flow diagram of the present study.

Data from 8 patients were excluded: 4 for incomplete chart records and 4 for simultaneous ridge augmentation during MSFA. Therefore, a total of 168 patients, corresponding to 182 maxillary sinuses, were included for the radiographic and histological analysis. Complete CBCT data were available for 154 maxillary sinuses and included for radiographic analysis. Specimens with insufficient tissue or damage were excluded from the histological analysis, leaving 147 native bone specimens and 95 grafted bone specimens for the histomorphometric analysis (Figure 3).

### Radiographic analysis

Among the 154 posterior maxillae, the mean RBH was 3.01 ± 2.05 mm (range: 0.50-4.00 mm). Based on the CBCT morphology, the sinuses were classified as:

• Type a—bicortical bone (i.e. both the sinus floor bone and the crestal bone are cortical bone): 65 sinuses (42.21%)
• Type b—unicortical bone (i.e. either the sinus floor bone or the crestal bone is cortical bone): 40 sinuses (25.97%).
• Type c—no cortical bone (i.e. neither the sinus floor bone nor the crestal bone is cortical): 30 sinuses (19.48%).
• Type d—no bone coronal to the sinus floor (i.e. fusion of the sinus floor bone and crestal bone): 19 sinuses (12.34%).

Inter-examiner agreement for CBCT sinus morphology classification was substantial (κ = 0.69; *P* < .01).

### Histomorphometric analysis

**Fig. 4:**
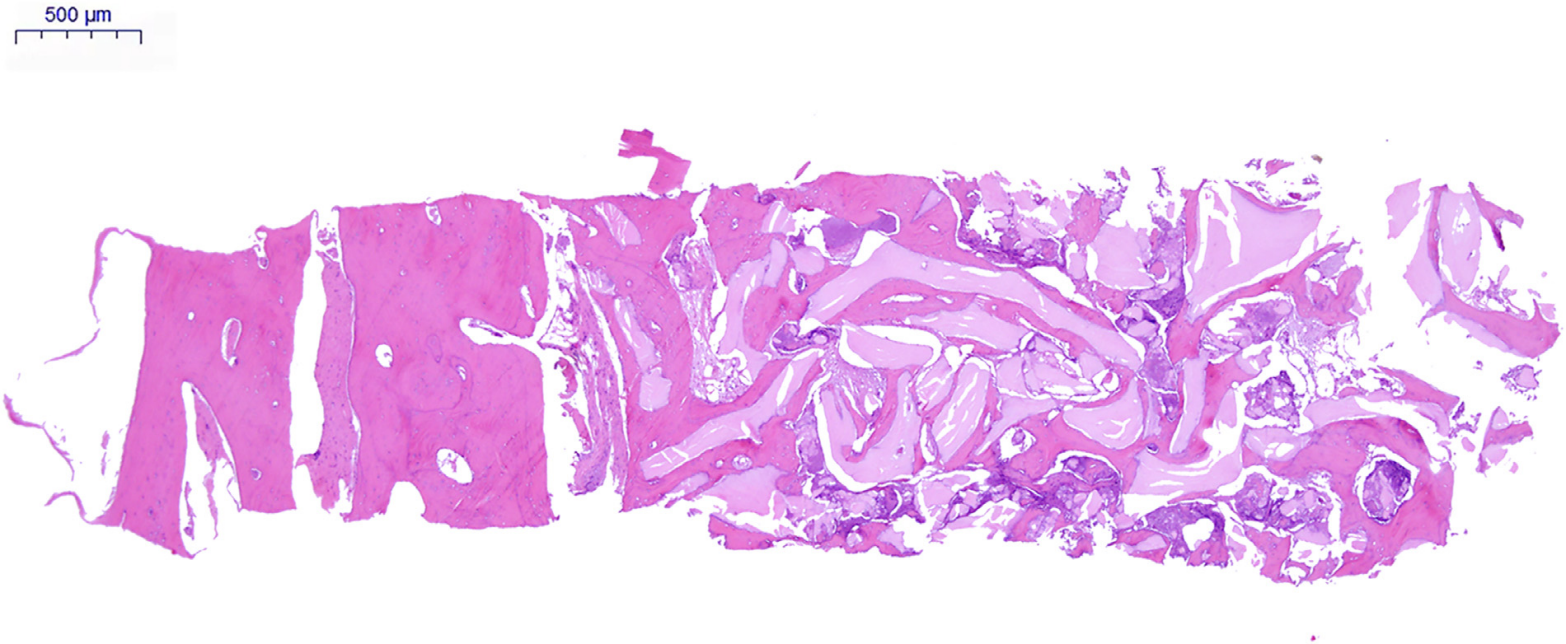
Observation image of the histological section under low magnification (×4). On the left side is the native bone area, mainly composed of mature trabeculae and bone marrow. On the right site is the grafted bone area, mainly composed of DBBM particles, newly formed bone and non-mineralised tissue.

**Fig. 5:**
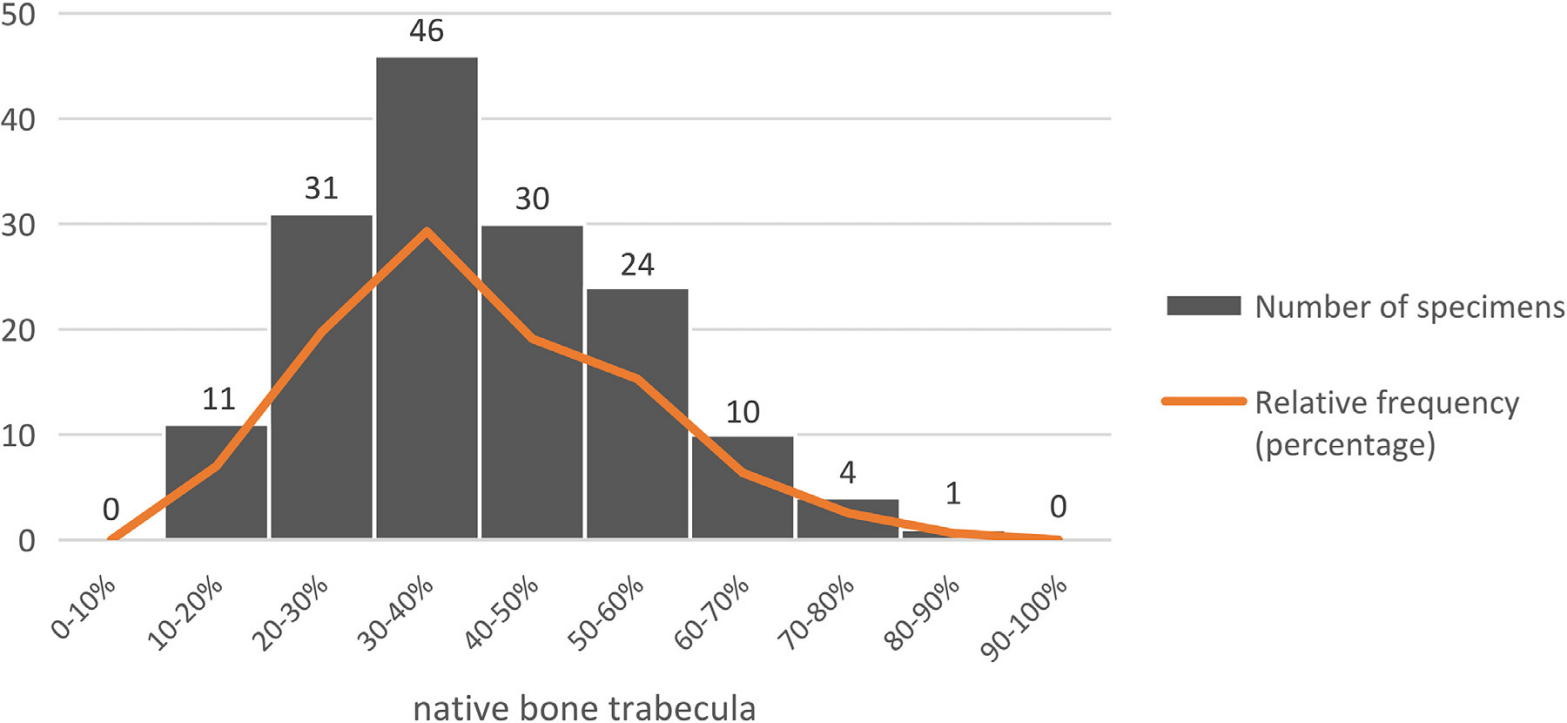
Distribution of native bone trabecula (%) in the resorbed posterior maxilla.

**Fig. 6:**
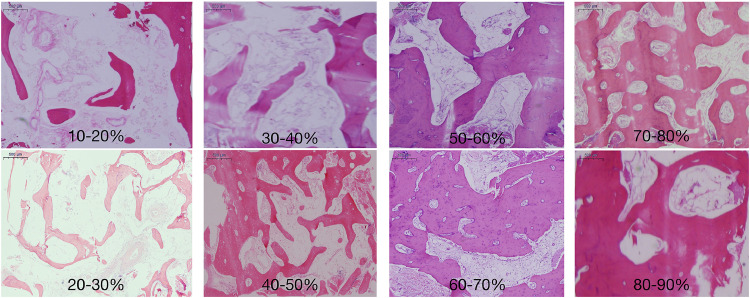
Representative histological images of the resorbed posterior maxilla with different native bone trabecula (%).

Native bone areas were mainly composed of mature trabeculae, characterised by mineralised lamellar bone and a randomly distributed pattern, and large bone marrow spaces infiltrated with loosely organised fibrous connective tissue, abundant adipocytes and small- to medium-diameter blood vessels (Figure 4). The mean native bone trabecula percentage was 40.01% ± 14.85% (range: 10.49%-82.56%). The most frequent native bone trabecula percentage ranges were 30%-40%, followed by 20%-30%, 40%-50% and 50%-60% (Figures 5 and 6). The inter-rater reliability for native bone trabecula percentage assessment was excellent (ICC = 0.99; *P* < .01)

In the grafted bone area, the DBBM particles appeared as pale pink structures with sharp edges, with empty osteocyte lacunae in the middle. Newly formed bone presented deep staining with a large number of osteocytes arranged in long strips, partly covering the surface of DBBM and bridging adjacent particles. The mean newly formed bone percentage was 18.77% ± 5.69% (range: 8.06%-31.13%), presenting also high inter-rater reliability (ICC = 0.98; *P* < .01).

### Correlation analysis

Correlation between the radiographic and histomorphometric data was conducted in 139 posterior maxillae. The native bone trabeculae percentages for the different types of alveolar ridges in the resorbed posterior maxilla are presented in Table 1. Although radiographic Type d presents an apparent sinus floor–crestal bone fusion, histological evaluation revealed a thin but continuous zone of mineralised bone beneath the fused cortical plate, allowing quantitative assessment of native bone trabeculae.

**Table 1:** Mean histomorphometric percentage of native bone trabecula of the biopsies related to the radiographic alveolar type of the resorbed posterior maxilla

Radiographic alveolar type	Number of biopsies	Histological native bone trabecula %		
		mean ± SD	minimum	maximum
Type a	57	38.29 ± 17.93	18.28	72.11
Type b	36	42.33 ± 16.18	10.49	82.56
Type c	29	38.84 ± 16.31	14.25	73.37
Type d	17	39.52 ± 15.21	13.70	59.99

Native bone trabeculae percentages did not differ significantly among the 4 types of radiographic bone morphology (*P* = .65). Neither gender (*P* = .69) nor age (*P* = .10) showed a statistically significant correlation with native bone trabecula percentages.

**Fig. 7:**
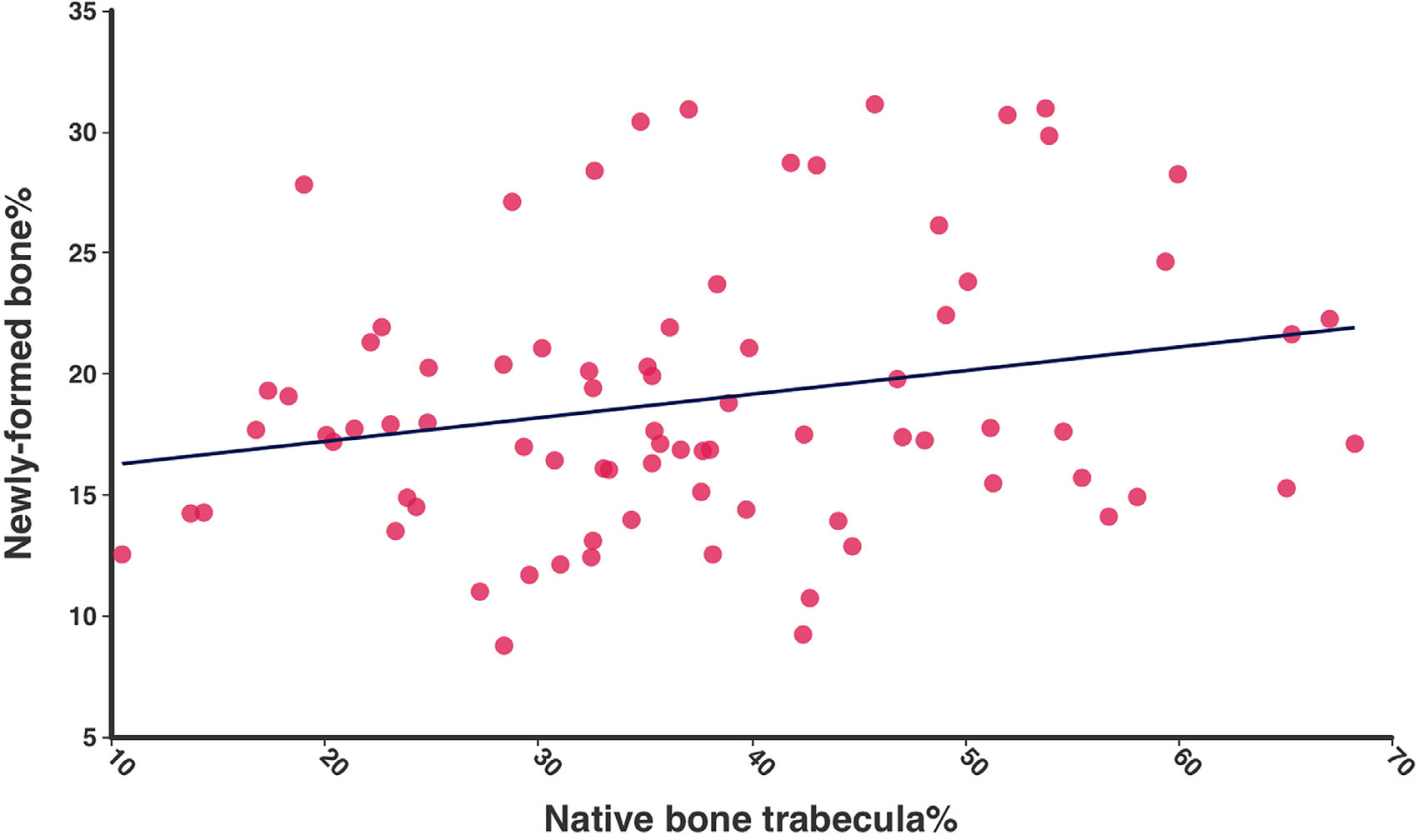
Scatter plot demonstrating the correlation between native bone trabecula (%) and newly formed bone (%).

Multiple linear regression revealed a positive correlation between native bone trabecula percentage and newly formed bone percentage (r = 0.23; *P* = .04; 95% CI = 0.01-0.19) (Figure 7). No statistically significant correlations were found between newly formed bone percentage and gender (*P* = .59), age (*P* = .70), healing time (*P* = .06) or radiographic bone type (*P* = .82).

## Discussion

Bone quality has long been recognised as a key determinant of dental implant success, with early studies associating implants placed in poor-quality bone with significantly higher failure rates.^18^^,^^19^ Although recent advancements in implant macro and micro design have enhanced osseointegration and the likelihood of achieving primary stability—thereby reducing the emphasis on bone quality—the thorough assessment of bone quality remains crucial for determining the potential treatment prognosis in challenging scenarios such as the severely resorbed posterior maxilla, where MSFA is needed before implant placement. In the present large retrospective study, we found a positive association between preoperative native bone quality—evaluated radiographically and histologically—and the subsequent new bone formation following MSFA, suggesting that posterior maxilla with denser residual ridge structure may favour osteogenesis.

Our radiographic analysis revealed that more than 40% of the alveolar ridge in the resorbed posterior maxilla exhibited a double layer of cortical bone, indicating a substantial remaining amount of cortical bone. The histomorphometric observations further showed that the native trabecular bone represented between 30% and 40% of the region of interest, closely aligning with Todisco and Trisi histologic study, reporting a mean bone volume percentage of 33.83% ± 10.92% in the maxillary premolar area and 30.62% ± 14.08% in the molar area.^20^ However, these values are based on the results of a small sample size, and the status of the alveolar bone (post-extraction or healed) was not described.^20^ Otherwise, Lindhe et al. with a relatively large sample size, reported the presence of 55.2% of mineralised bone at fully healed sites in the posterior maxilla,^13^^,^^14^ exceeding our findings. This discrepancy likely reflects the inverse correlation between RBH in the posterior maxilla and trabecular bone quality and quantity, give that previous works show that alveolar bone mineralisation decreases with the reduction in bone volume.^21^

Traditional radiographical classifications of alveolar bone quality, such as Lekholm and Zarb’s four types, remain one of the most widely used methods worldwide. In fact, for the posterior maxilla, bone quality frequency has been reported as type III in 73.33%, type II in 13.33% and type IV in 13.33% of cases.^12^ However, its application was problematic when the cortical bone was discontinuous, and when distinguishing between dense trabecular bone (type III) and low-density bone (type IV). Instead, the use of the modified classification method by Choucroun et al.,^17^ based on remaining cortical bone, demonstrated superior inter-examiner agreement by providing a more objective quantitative criteria for preoperative bone quality assessment.

Extensive angiogenesis and osteoblastic migration from the native bone walls are fundamental for osteogenesis at the maxillary sinus regardless of the type of grafted material, eventually being transformed into new bone by "creeping substitution."^22^^,^^23^ However, whether the quality of the native bone wall influences the osteogenic outcome of the grafted area is still inconclusive. In this context, Reich et al. evaluated the bone volume fraction of sinus biopsies and found a positive correlation between the native and grafted bone quality,^24^ consistent with the results of the present study. In addition, a recent study reported a positive correlation between bone quality and bone regeneration volume after reduction mandibuloplasty, concluding that higher density of surrounding bone benefits local bone regeneration and growth.^25^ Therefore, altogether our results also support the hypothesis that denser native bone—with greater osteoblast reservoirs and stronger structural support—promotes a positive bone grafting outcome, whereas poor native bone quality—with less bone matrix and being more porous—may compromise graft integration and maturation or result in less predictable osteogenesis. Nevertheless, further research is required to determine how the composition of the native recipient bone affects osteogenesis in grafted areas.

Bone quality was evaluated primarily from a histomorphometric and structural standpoint in the present study, using the proportion of mineralised trabecular bone as a surrogate parameter. This measurement reflects bone quantity and microstructural organisation rather than bone mineral density per se, which requires dedicated densitometric techniques. Moreover, vascularity is a critical biological component of bone quality and may further influence osteogenesis. However, quantitative assessment of vascular parameters was beyond the scope of this retrospective analysis and was not performed. Future studies incorporating vascular and densitometric assessments may provide a more comprehensive characterisation of bone quality.

CBCT slices and histological sections provide valuable information on bone structure, but both assessments are inherently 2-dimensional and therefore cannot fully reflect the 3-dimensional complexity of the sinus floor. Future studies incorporating 3-dimensional radiographic reconstruction or micro-CT may further clarify the spatial characteristics of the recipient bone. The primary limitation of this study lies in its retrospective design, which may inherently allow confounding factors that may influence the osteogenesis outcomes following MSFA. Although our regression models found no significant effects of patient age, gender or healing time, the histomorphometric findings indicated that osteogenesis after MSFA was significantly influenced by the quality of the alveolar bone beneath the sinus floor. Apart from that, the healing times for the cases included in this study varied substantially, ranging from 4 to 12 months. Nonetheless, a prior prospective study by our group showed no differences in newly formed bone regardless of healing time (5, 7, and 11 months post-surgery) after MSFA,^26^ thus aligning with the results observed in our multiple linear regression model in the present study. Otherwise, anatomical morphology of the maxillary sinus can also play a role in internal osteogenesis. For instance, the width of the sinus floor has been negatively correlated with new bone formation,^16^^,^^27^, ^28^, ^29^, ^30^, ^31^ whereas RBH has not shown a significant effect.^16^^,^^32^ While other factors, such as surgical techniques and the choice of graft materials, may also influence the osteogenesis process,^33^, ^34^, ^35^ it was not feasible to account for all variables within the regression model. Consequently, this study focused its correlation analysis on baseline variables and those specifically related to alveolar bone quality.

The present study focused on the posterior maxilla and MSFA as the primary research model; however, the concept that the quality of the native recipient bone influences subsequent osteogenesis may also be applicable to other bone-grafting procedures, such as guided bone regeneration (GBR), onlay bone grafting and socket preservation. In these procedures, the native bone walls serve as the principal source of vascular supply, osteogenic cells and biological signalling. Therefore, a denser and more structured recipient bone may facilitate faster graft integration and more predictable bone maturation, whereas compromised native bone quality may necessitate modified surgical strategies, prolonged healing periods or staged treatment approaches. From a clinical perspective, incorporating preoperative assessment of native bone quality into treatment planning may help clinicians to better estimate regenerative potential, optimise surgical protocols and improve prognostic predictability across various bone augmentation procedures. To better understand the impact of recipient site bone quality on osteogenesis following bone augmentation, future research should include well-designed prospective studies. This will help to clarify the role of bone quality in optimising clinical outcomes across various bone augmentation techniques.

## Conclusions

In this large cohort of resorbed posterior maxilla, more than 40% of the alveolar ridges had a double layer of cortical bone, and nearly one-third exhibited a proportion of mineralised bone trabecula between 30% and 40%. Denser alveolar bone quality was positively associated with new bone formation after MSFA, suggesting that preoperative assessment of native bone density can help to predict—and potentially improve—osteogenic outcomes in MSFA procedures.

## Author contributions

*Conception and design:* Fan, Wu, Zhou; *Data acquisition:* Shen, Zhou; *Analysis and interpretation:* Cafferata, Fan, Shen, Wu, Zhou; *Surgery execution:* Wu; *Funding:* Wu; Writing—original draft: drafted the manuscript. Zhou; *Writing—editing and revision:* Cafferata, Fan, Wu.

## Funding

This work was supported by the “Multidisciplinary Team” Clinical Research Project of the Ninth People’s Hospital, affiliated to the Shanghai Jiao Tong University, School of Medicine (2017-1-005), CAMS Innovation Fund for Medical Sciences (CIFMS) (2019-I2M-5-037), Clinical Research Plan of SHDC (SHDC2020CR3049B) and Research Discipline fund (KQYJXK2020) from the Ninth People’s Hospital, Shanghai Jiao Tong University School of Medicine and College of Stomatology, Shanghai Jiao Tong University.

## Conflict of interests

None declared.

## Appendix Supplementary materials

Image, application 1

Image, image 2 dummy alt text

Image, application 3
