# Supplementary material for: Bone Quality in Resorbed Posterior Maxilla Affects Osteogenesis After Sinus Floor Augmentation: A Retrospective Analysis
Source: Int Dent J. 2026 Jan 30;76(2):109397. doi: 10.1016/j.identj.2025.109397 (PMC12876791; doi:10.1016/j.identj.2025.109397)
Supplement: Supplementary file 1 [file mmc1.doc]

STROBE Statement—Checklist of items that should be included in reports of ***cross-sectional studies***

|  | Item No | Recommendation | Page  No | Relevant text from manuscript |
| --- | --- | --- | --- | --- |
| **Title and abstract** | 1 | (*a*) Indicate the study’s design with a commonly used term in the title or the abstract | Title page | Bone quality in resorbed posterior maxilla affects osteogenesis after sinus floor augmentation: A retrospective analysis |
|  |  | (*b*) Provide in the abstract an informative and balanced summary of what was done and what was found | 1 | **Materials and Methods:** Patients with advanced posterior maxillary atrophy (≤ 4 mm residual bone height) underwent MSFA using deproteinized bovine bone matrix (DBBM) via a lateral approach. Bone core specimens were collected during second-stage implant placement for histological analyses. Preoperative CBCT images were used to classify bone quality based on cortical bone presence. Multiple linear regression analyzed factors influencing osteogenesis.  **Results:** Between 2015 and 2021, 190 sinuses of 176 patients (96 males and 80 females, age 45.77 ± 4.13 years) underwent augmentation and biopsy. Radiographical observation showed that 42.21% of maxillary ridges had bicortical bone, 25.97% had unicortical bone, 19.48% had no cortical bone, and 12.34% showed fusion of sinus floor and crestal bone. Histologically, native bone comprised 40.01% ± 14.85% mineralized trabeculae, and grafted areas showed 18.77% ± 5.69% of newly-formed bone. A positive correlation was found between native bone trabecula percentage and new bone formation (r = 0.23, *p* = 0.04), while no significant correlations were noted with age, gender, healing time, or radiographic alveolar type ( *p* > 0.05). |
| Introduction | | |  |  |
| Background/rationale | 2 | Explain the scientific background and rationale for the investigation being reported | 2 | The compromised bone quality and quantity have been consistently considered as one of the major risk factors for implant failures in this region, highlighting the importance of pretreatment assessment of potential implant sites to achieve favorable treatment prognosis.  Therefore, the study on alveolar bone quality is critical for clinical decision-making, for example implant placement and loading protocols, implant selection and guidance approach for comuputer-guided surgery.  However, there is a lack of studies specifically demonstrating the bone quality in the resorbed posterior maxilla, and few research has investigated whether the bone quality of the recipient site influences osteogenesis following maxillary sinus floor augmentation (MSFA). |
| Objectives | 3 | State specific objectives, including any prespecified hypotheses | 3 | Therefore, the study aimed to assess the bone quality of the resorbed posterior maxilla both radiographically and histologically, and to investigate the effect of native bone quality on osteogenesis after MSFA. |
| Methods | | |  |  |
| Study design | 4 | Present key elements of study design early in the paper | 3 | The study protocol was approved by the Institutional Review Board of Shanghai Ninth People's Hospital, China (No: 2015[78] and SH9H-2021-T63-1). The study reporting was adhere to the checklist items of the Strengthening the Reporting of Observational Studies in Epidemiology (STROBE) guidelines. |
| Setting | 5 | Describe the setting, locations, and relevant dates, including periods of recruitment, exposure, follow-up, and data collection | 3 | All patients included in this study were referred to the Department of Oral Implantology or the Second Dental Center, Ninth People’s Hospital, Shanghai Jiao Tong University, School of Medicine for implant treatment in the resorbed posterior maxilla from June 2015 to September 2021. |
| Participants | 6 | Give the eligibility criteria, and the sources and methods of selection of participants | 3 | Eligible patients were adults (≥ 18 years) presenting maxillary molar(s) or premolar(s) loss ≥3 months, and residual bone height (RBH) ≤ 4 mm and adequate bone width at target edentulous site, providing signed informed consent for participation. Exclusion criteria included active sinus infection, uncontrolled inflammatory diseases – e.g. uncontrolled periodontal disease, uncontrolled diabetes, heavy smoking (> 10 cigarettes/day), history of head/neck radiation therapy (> 60 Gy doses), osteoporosis or bisphosphonates/steroids therapy, and incomplete records. |
| Variables | 7 | Clearly define all outcomes, exposures, predictors, potential confounders, and effect modifiers. Give diagnostic criteria, if applicable | 4 | Bone quality of the maxillary ridge was evaluated and classified according to a modified category after Choucroun et al.16 (Figure 1). |
| Data sources/ measurement | 8* | For each variable of interest, give sources of data and details of methods of assessment (measurement). Describe comparability of assessment methods if there is more than one group | 4 | CBCT data were collected within one month before MSFA. CBCT images were obtained using the Planmeca Promax Tomography System (Planmeca USA, Inc., Chicago, USA) with the following operating parameters: 5 mA, 96 kV; voxel size: 0.2 mm; field of view: 13 cm × 9 cm.  The primary data were exported as DICOM files and reconstructed using an implant planning software (coDiagnostiX®, Dental Wings GmbH, Chemnitz, Germany). For each case, a digital wax-up and surgical planning were performed prior to the surgery, following the methodology described by Zhou et al., 2021.15 Measurements were taken from the sagittal sections at the implant site in accordance with the prosthetic planning. RBH was measured as the vertical distance from the alveolar crest to the sinus floor, along the long axis of the virtual implant. Bone quality of the maxillary ridge was evaluated and classified according to a modified category after Choucroun et al.16 (Figure 1).  These images were imported into Image Pro-Plus software (version 6.0, Media Cybernetics, Silver Spring, MD, USA) for quantitative analysis. The regions of interest—the native bone trabeculae in the native bone area and the newly formed bone in the grafted bone area—were automatically delineated by the software based on color contrast and subsequently calibrated manually. The percentage of native bone trabeculae and newly formed bone was quantified by calculating the ratio of selected pixels to total pixel counts. The results were averaged across independent measurements from the three sections. |
| Bias | 9 | Describe any efforts to address potential sources of bias | 4 | The primary data were exported as DICOM files and reconstructed using an implant planning software (coDiagnostiX®, Dental Wings GmbH, Chemnitz, Germany).  For each patient, a digital wax-up and surgical planning were performed prior to the surgery, following the protocol described by Zhou et al., 202116. Radiographic measurements were taken from sagittal sections at the planned implant sites, according to the pre-prosthetic planning. RBH was defined as the vertical distance from the alveolar crest to the sinus floor, measured along the long axis of the virtual implant. The bone quality of the maxillary ridge was assessed and classified using a modified version of the classification proposed by Choucroun et al.17 (Figure 1).  The results were averaged across independent measurements from the three sections. |
| Study size | 10 | Explain how the study size was arrived at | 3 | All patients referred to the Department of Oral Implantology or the Second Dental Center, Ninth People’s Hospital, Shanghai Jiao Tong University, School of Medicine, between June 2015 to September 2021, with indication for implant rehabilitation of the atrophic posterior maxilla were consecutively screened for eligibility. |
| Quantitative variables | 11 | Explain how quantitative variables were handled in the analyses. If applicable, describe which groupings were chosen and why | NA |  |
| Statistical methods | 12 | (*a*) Describe all statistical methods, including those used to control for confounding | 5 | The inter-examiner agreement was assessed with the Kappa test for categorical variables (radiographical alveolar type). The inter-rater reliability was determined with the interclass correlation coefficient (ICC) for continuous variables (% of native bone trabeculae and % of newly-formed bone). All measurements were analyzed at the sinus- and biopsy-levels.  To investigate the association between radiographic and histomorphometric outcomes of bone quality, one-way ANOVA was used to analyze the mean % of native bone trabecula from the specimens with the the radiographic classification of the resorbed posterior maxilla. A multiple linear regression model, with r values as correlation coefficient, assessed the associations between histomorphometric outcomes -mean % of native bone trabecula and newly-formed bone- , healing time and radiographic alveolar type. Generalized Estimating Equations (GEE) analysis was performed using baseline variables (age and gender) as predictors for % of newly-formed bone, while adjusting for patients who had bilateral MSFA. Age (continuous) and gender (binary) were included as fixed effects, and an exchangeable correlation structure was used to model the relationship between surgeries on the same patient. Statistical significance was set at p < 0.05. All analyses were performed with the PASW Statistics 18.0 software program. |
|  |  | (*b*) Describe any methods used to examine subgroups and interactions | 5 | To investigate the association between radiographic and histomorphometric outcomes of bone quality, one-way ANOVA was used to analyze the mean % of native bone trabecula from the specimens with the the radiographic classification of the resorbed posterior maxilla. A multiple linear regression model, with r values as correlation coefficient, assessed the associations between histomorphometric outcomes -mean % of native bone trabecula and newly-formed bone- , healing time and radiographic alveolar type. Generalized Estimating Equations (GEE) analysis was performed using baseline variables (age and gender) as predictors for % of newly-formed bone, while adjusting for patients who had bilateral MSFA. Age (continuous) and gender (binary) were included as fixed effects, and an exchangeable correlation structure was used to model the relationship between surgeries on the same patient. Statistical significance was set at p < 0.05. All analyses were performed with the PASW Statistics 18.0 software program. |
|  |  | (*c*) Explain how missing data were addressed | NA |  |
|  |  | (*d*) If applicable, describe analytical methods taking account of sampling strategy | NA |  |
|  |  | (*e*) Describe any sensitivity analyses | NA |  |
| Results | | |  |  |
| Participants | 13* | (a) Report numbers of individuals at each stage of study—eg numbers potentially eligible, examined for eligibility, confirmed eligible, included in the study, completing follow-up, and analysed | 6 and Figure 2 | Data from 8 patients were excluded due to the following reasons: 4 for incomplete chart records, and 4 for simultaneous ridge augmentation during MSFA. Therefore, a total of 168 patients, corresponding to 182 maxillary sinuses, were included for the radiographic and histological analysis. Complete CBCT data were available for 154 maxillary sinuses and included for radiographic analysis. Specimens with insufficient tissue or damage were excluded from the histological analysis, leaving 147 native bone specimens and 95 grafted bone specimens for the histomorphometric analysis (Figure 2). |
|  |  | (b) Give reasons for non-participation at each stage | 6 | Data from 8 patients were excluded due to the following reasons: 4 for incomplete chart records, and 4 for simultaneous ridge augmentation during MSFA. |
|  |  | (c) Consider use of a flow diagram | Figure 2 |  |
| Descriptive data | 14* | (a) Give characteristics of study participants (eg demographic, clinical, social) and information on exposures and potential confounders | 6 | A total of 176 patients, corresponding to 190 maxillary sinuses, underwent MSFA via a lateral approach, with bone biopsies obtained at the implant sites. The study sample comprised 96 males and 80 females, (mean age: 45.77 ± 4.13 years; range: 20–72 years). The mean healing time after MSFA was 8.14 ± 4.77 months (range: 4.0–12.4 months). |
|  |  | (b) Indicate number of participants with missing data for each variable of interest | 6 | A total of 176 patients, corresponding to 190 maxillary sinuses, underwent MSFA via a lateral approach, with bone biopsies obtained at the implant sites. |
| Outcome data | 15* | Report numbers of outcome events or summary measures | 6 | Complete CBCT data were available for 154 maxillary sinuses and included for radiographic analysis. Specimens with insufficient tissue or damage were excluded from the histological analysis, leaving 147 native bone specimens and 95 grafted bone specimens for the histomorphometric analysis (Figure 2). |
| Main results | 16 | (*a*) Give unadjusted estimates and, if applicable, confounder-adjusted estimates and their precision (eg, 95% confidence interval). Make clear which confounders were adjusted for and why they were included | 6-7 | Among the 154 posterior maxillae, the mean RBH was 3.01 ± 2.05 mm (range: 0.50–4.00 mm). Based on the CBCT morphology, sinuses were classified as:  Type a -bicortical bone- (i.e. both the sinus floor bone and the crestal bone are cortical bone): 65 sinuses (42.21%)  Type b -unicortical- (i.e. either the sinus floor bone or the crestal bone is cortical bone):  40 sinuses (25.97%).  Type c -no cortical bone- (i.e. neither the sinus floor bone nor the crestal bone is cortical):  30 sinuses (19.48%).  Type d -no bone coronal to the sinus floor- (i.e. fusion of the sinus floor bone and crestal bone): 19 sinuses (12.34%).  Inter-examiner agreement for CBCT sinus morphology classification was substantial ( = 0.69; p < 0.01).  The mean native bone trabecula percentage was 40.01% ± 14.85% (range: 10.49%–82.56%). The most frequent native bone trabecula % ranges were 30–40%, followed by 20–30%, 40–50% and 50–60% (Figure 4 and 5). The inter-rater reliability for native bone trabecula % assessment was excellent (ICC = 0.99; p < 0.01)  The mean newly-formed bone percentage was 18.77% ± 5.69% (range: 8.06%–31.13%), presenting also high inter-rater reliability (ICC = 0.98; *p* < 0.01). |
|  |  | (*b*) Report category boundaries when continuous variables were categorized | NA |  |
|  |  | (*c*) If relevant, consider translating estimates of relative risk into absolute risk for a meaningful time period | NA |  |
| Other analyses | 17 | Report other analyses done—eg analyses of subgroups and interactions, and sensitivity analyses | 7 | Correlation between the radiographic and histomorphometric data was conducted in 139 posterior maxillae. The native bone trabeculae percentages for the different types of alveolar ridges in the resorbed posterior maxilla are presented in Table 1.  Native bone trabeculae percentages did not differ significantly among the 4 types of radiographic bone morphology (p = 0.65). Neither gender (p = 0.69) nor age (p = 0.10) showed a statistically significant correlation with native bone trabecula%.  Multiple linear regression revealed a positive correlation between native bone trabecula % and newly-formed bone % (r = 0.23; p = 0.04; 95% CI = 0.01–0.19) (Figure 6). No statistically significant correlations were found between newly-formed bone% and gender (p = 0.59), age (p = 0.70), healing time (p = 0.06), or radiographic bone type (p = 0.82). |
| Discussion | | |  |  |
| Key results | 18 | Summarise key results with reference to study objectives | 8-9 | Our radiographic analysis revealed that over 40% of the alveolar ridge in the resorbed posterior maxilla exhibited a double layer of cortical bone, indicating a substantial remaining of cortical bone. Moreover, the histomorphometric observations further showed that the native trabecular bone represented between 30% and 40% of the region of interest.  Although our regression models found no significant effects of patient age, gender, or healing time, the histomorphometric findings indicated that osteogenesis after MSFA was significantly influenced by the quality of the alveolar bone beneath the sinus floor. |
| Limitations | 19 | Discuss limitations of the study, taking into account sources of potential bias or imprecision. Discuss both direction and magnitude of any potential bias | 9 | The primary limitation of this study lies in its retrospective design, which may inherently allow confounding factors that may influence the osteogenesis outcomes following MSFA. Although our regression models found no significant effects of patient age, gender, or healing time, the histomorphometric findings indicated that osteogenesis after MSFA was significantly influenced by the quality of the alveolar bone beneath the sinus floor. Apart from that, the healing times for the cases included in this study varied substantially, ranging from 4 to 12 months. Nonetheless, a prior prospective study by our group showed no differences in newly-formed bone regardless of healing time (5, 7, and 11 months post-surgery) after MSFA26, thus aligning with the results observed in our multiple linear regression model in the present study. Otherwise, anatomical morphology of the maxillary sinus can also play a role in internal osteogenesis. For instance, the width of the sinus floor has been negatively correlated with new bone formation16, 27–31, while RBH has not shown a significant effect16, 32. While other factors, such as surgical techniques and the choice of graft materials, may also influence the osteogenesis process33–35, it was not feasible to account for all variables within the regression model. Consequently, this study focused its correlation analysis on baseline variables and those specifically related to alveolar bone quality. To better understand the impact of recipient site bone quality on osteogenesis following bone augmentation, future research should include well-designed prospective studies. This will help clarify the role of bone quality in optimizing clinical outcomes across various bone augmentation techniques. |
| Interpretation | 20 | Give a cautious overall interpretation of results considering objectives, limitations, multiplicity of analyses, results from similar studies, and other relevant evidence | 10 | More than 40% of the alveolar ridge in the resorbed posterior maxilla had a double layer of cortical bone, and nearly one-third had a proportion of mineralized bone trabecula between 30% to 40%. Bone quality of the posterior maxilla has a positive effect on the osteogenic results after MSFA, indicating that a denser bone structure can be a contributing factor for better osteogenesis. |
| Generalisability | 21 | Discuss the generalisability (external validity) of the study results | 10 | The present study focused on the posterior maxilla and MSFA as the research objects. The method of this study is also applicable to other bone grafting procedures such as guided bone regeneration (GBR), onlay bone grafting, socket preservation, etc. |
| Other information | | |  |  |
| Funding | 22 | Give the source of funding and the role of the funders for the present study and, if applicable, for the original study on which the present article is based | 10 | This work was supported by the “Multidisciplinary Team” Clinical Research Project of the Ninth People’s Hospital, affiliated to the Shanghai Jiao Tong University, School of Medicine (2017-1-005), CAMS Innovation Fund for Medical Sciences (CIFMS) (2019-I2M-5-037), Clinical Research Plan of SHDC (SHDC2020CR3049B), and Research Discipline fund (KQYJXK2020) from the Ninth People’s Hospital, Shanghai Jiao Tong University School of Medicine, and College of Stomatology, Shanghai Jiao Tong University. |

*Give information separately for exposed and unexposed groups.

**Note:** An Explanation and Elaboration article discusses each checklist item and gives methodological background and published examples of transparent reporting. The STROBE checklist is best used in conjunction with this article (freely available on the Web sites of PLoS Medicine at http://www.plosmedicine.org/, Annals of Internal Medicine at http://www.annals.org/, and Epidemiology at http://www.epidem.com/). Information on the STROBE Initiative is available at www.strobe-statement.org.
