# Supplementary figures and images for: Bone Quality in Resorbed Posterior Maxilla Affects Osteogenesis After Sinus Floor Augmentation: A Retrospective Analysis
Source: Int Dent J. 2026 Jan 30;76(2):109397. doi: 10.1016/j.identj.2025.109397 (PMC12876791; doi:10.1016/j.identj.2025.109397)

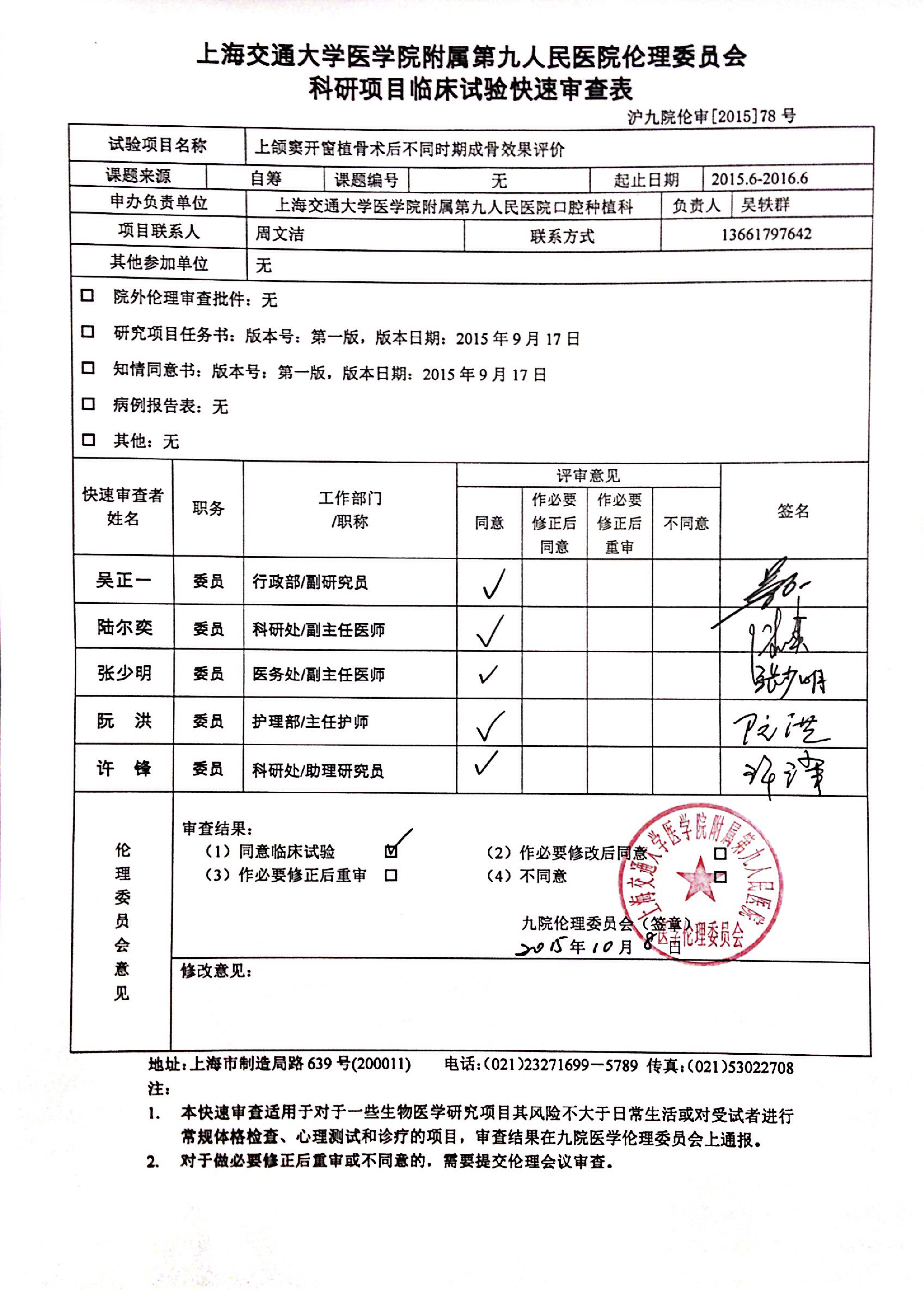

Supplement: Supplementary file 2 [file mmc2.jpg]
