# Supplementary material for: Bone Quality in Resorbed Posterior Maxilla Affects Osteogenesis After Sinus Floor Augmentation: A Retrospective Analysis
Source: Int Dent J. 2026 Jan 30;76(2):109397. doi: 10.1016/j.identj.2025.109397 (PMC12876791; doi:10.1016/j.identj.2025.109397)
Supplement: Supplementary file 3 [file mmc3.pdf]

上海交通大学医学院附属第九人民医院医学伦理委员会

伦理审查批准函

声明：伦理委员会按照国家卫健委有关法规组成和工作，其审查和工作过程不受伦理委员会以外任何组织及个人的影响

批件号：SH9H-2021-T63-1

研究项目名称	无机脱蛋白牛骨经外侧壁开窗植入上颌窦底的成骨作用
审查会议地点	8 号楼 301
申办方	口腔第二门诊部
研究单位	上海交通大学医学院附属第九人民医院
主要研究者	周文洁
伦理审查方式	■会议审查　　第(1)次会议审查时间：2021-04-29<br>□快速审查
审查文件	初始审查清单：<br>1.临床试验方案　　版本号：20210220，版本日期：2021 年 02 月 20 日<br>2.知情同意书　　版本号：20210220，版本日期：2021 年 02 月 20 日<br>3.研究者简历　　日期：2021 年 02 月 20 日<br>4.研究者声明　　日期：2021 年 02 月 20 日
审查意见	1.　经伦理委员会审查，同意该项临床研究。<br>2.伦理委员会对该研究实施过程的持续跟踪审查：■是　□否<br>审查频度为：□　3 个月　□　6 个月　■　12 个月　□不改变<br>3.伦理委员会有权根据实际进展情况改变持续跟踪审查频度。<br>4.自批准之日起一年内项目未启动，该批件自动失效。

主任或副主任委员签字：

伦理委员会（盖章）

日期：2021.5.7

上海交通大学医学院附属第九人民医院 转化医学伦理审查专委会

注意：（请仔细阅读）

1.伦理委员会批准的项目为涉及人体的生物医学研究，必须严格按照所批最新版本的研究方案和知情同意书开展研究，并遵循国内相关法规指南要求。

2.凡是涉及人类遗传资源出口或者按照国家规定必须经有关部门专项审批的内容，均需在项目执行前向有关部门申报并获得批准。

3.本批件可能用于其他中心伦理委员会参考，如果对审查存在不同意见，请及时与本伦理委员会沟通。

4.对已批准的研究方案、知情同意书等材料的任何修改及主要研究者更换等，须及时通知本伦理委员会重新审查，获得批准后执行。

5.发生严重不良事件及影响研究风险受益比的非预期事件，须及时报告本机构伦理委员会。

6. 根据伦理委员会对年度/定期跟踪审查频度的意见，无论研究开始与否，请在年度/定期跟踪审查日到期前 1 个月提出年度/定期跟踪审查的申请。

7.发现不依从/违反方案情况须及时报告伦理委员会审查。

8.暂停/提前终止临床研究，请及时通知伦理委员会。

9.完成研究，须提交结题报告供伦理委员会审查。

地址：上海市制造局路 639 号(200011)

电话：（021）23271699－5576

传真：（021）63057795

上海交通大学医学院附属第九人民医院

转化医学伦理审查专委会

签到及声明

会议时间：2021 年 04 月 29 日　　　　**会议地点：8 号楼 301**

姓　名	职　务	专　业	性别	签名	日期
罗　蒙	主　任	外科学	男	罗蒙	2021.4.29
王　艳	副主任	预防医学	女	王艳	
姚　敏	副主任	外科学	女	姚敏	2021.4.29
冯希平	委　员	口腔医学	男	冯希平	2021-4-29
谢幼专	委　员	外科学	男		
陆颖理	委　员	内科学	女	陆颖理	2021-4-29
顾　岩	委　员	外科学	男	顾岩	2021.4.29
何　悦	委　员	口腔医学	男	何悦	2021.4.29
刘　凯	委　员	外科学	男	刘凯	2021-4-29
姜　虹	委　员	麻醉学	女	姜虹	
丁　峰	委　员	内科学	男	丁峰	2021.4.29
谷　平	委　员	眼科学	男	谷平	2021.4.29
许　锋	委　员	外科学、临床研究管理	女	许锋	2021-4-29
邵　斌	委　员	外 院	男		
胡庆澧	委　员	伦理学	男		
施强华	委　员	社会学	男	施强华	2021.4.29
赵耐青	委　员	统计学	男		
祝明真	委　员	法　学	女	祝明真	祝 2021.4.29
甄　红	秘　书	生物工程	女		
刘墨池	秘　书	社会学	女	刘墨池	2021.4.29

伦理委员会声明：

上海交通大学医学院附属第九人民医院医学伦理委员会的组成及工作程序遵循 ICH-GCP 原则和中国相关法律法规。
